# Vibration Energy Harvester Based on Torsionally Oscillating Magnet

**DOI:** 10.3390/mi12121545

**Published:** 2021-12-12

**Authors:** Xinyi Wang, Jiaxing Li, Chenyuan Zhou, Kai Tao, Dayong Qiao, Yunjia Li

**Affiliations:** 1 School of Electrical Engineering, Xi’an Jiaotong University, Xi’an 710049, China; xywang@stu.xjtu.edu.cn (X.W.); twinklestar2@stu.xjtu.edu.cn (J.L.); ZhouChenyuan@stu.xjtu.edu.cn (C.Z.); 2 Micro and Nano Electromechanical Systems Laboratory, Northwestern Polytechnical University, Xi’an 710072, China; taokai@nwpu.edu.cn (K.T.); dyqiao@nwpu.edu.cn (D.Q.)

**Keywords:** electromagnetic, energy harvester, MEMS, planar coil

## Abstract

Most of the miniaturized electromagnetic vibrational energy harvesters (EVEHs) are based on oscillating proof mass suspended by several springs or a cantilever structure. Such structural feature limits the miniaturization of the device’s footprint. This paper presents an EVEH device based on a torsional vibrating magnet over a stack of flexible planar coils. The torsional movement of the magnet is enabled by microfabricated silicon torsional springs, which effectively reduce the footprint of the device. With a size of 1 cm × 1 cm × 1.08 cm, the proposed EVEH is capable of generating an open-circuit peak-to-peak voltage of 169 mV and a power of 6.9 μW, under a sinusoidal excitation of ±0.5 g (g = 9.8 m/s^2^) and frequency of 96 Hz. At elevated acceleration levels, the maximum peak-to-peak output voltage is 222 mV under the acceleration of 7 g (±3.5 g).

## 1. Introduction

Vibration energy harvesters (VEHs) are devices that transduce vibration kinetic energy to electric power [1]. They have attracted much attention lately due to their potential as alternative power sources to batteries in vibration environments [2]. The vibration energy harvesters can be implemented with different operation principles, such as piezoelectric [3], electromagnetic [4], electrostatic [5], and triboelectric [6]. Among them, electromagnetic vibration energy harvesters (EVEHs) have the advantage of easy design and fabrication, relatively high output voltage and power, as well as good reliability [7]. The transduction of the EVEH is often realized by vibration-induced movement between a permanent magnet and a set of solenoid coils.

Traditional EVEHs can be implemented by fixing a macroscopic magnet or magnet array to the vicinity of a coil, which is a simple and robust approach [8]. However, the macroscopic EVEH is too bulky to power most of the smart electronic devices, giving rise to the strong demand to miniaturize the EVEHs. The typical methods to miniaturize the EVEHs are based on printed circuit board (PCB) [9,10,11] and microelectromechanical system (MEMS) technologies [12,13,14,15], where the coils are often fabricated with deposited planar coils. The main bottleneck for the planar coils is the limited number of turns, as the wires are confined within a single plane [16]. To cope with this limitation, the microfabricated vibrating proof mass is sometimes used with wound coils [17,18] However, such an approach has two issues: Firstly, the assembly process of the microcomponents is difficult; secondly, when the size of the coil is big, many of the turns in the coils cannot effectively cut the magnetic field generated with the micro magnet. In order to deal with these two issues, the authors have proposed a method based on stacked flexible coils, where high-density coils can be formed within the thickness range of 1–2 mm [19]. The low-cost repeating layers of flexible coils also simplifies the assembly and fabrication processes. It has been shown that with flexible coils, the EVEH device was capable of generating an output voltage of 1.56 V, with a volume of 7.5 cm^3^.

Toward the goal of miniaturization, the fabrication of the vibrating proof mass has been shifted from PCB to MEMS technologies in a previous study [20]. In these designs, the permanent magnet is suspended by four folded springs, which is a classic type of layout for reducing the resonant frequency of MEMS structures (in addition to cantilever structures). However, it is discovered that these design strategies limit the miniaturization of the device. Therefore, a structure with a torsionally oscillating magnet is proposed in 2021, which has been proven to be as effective as the folded spring design in terms of output power, but with a relatively small footprint [21]. In this work, we will extend our conference contributions in [21] and systematically present the design, implementation, and results of a torsional EVEH device.

## 2. System Design

### 2.1. Structure of the EVEH

**Figure 1:**
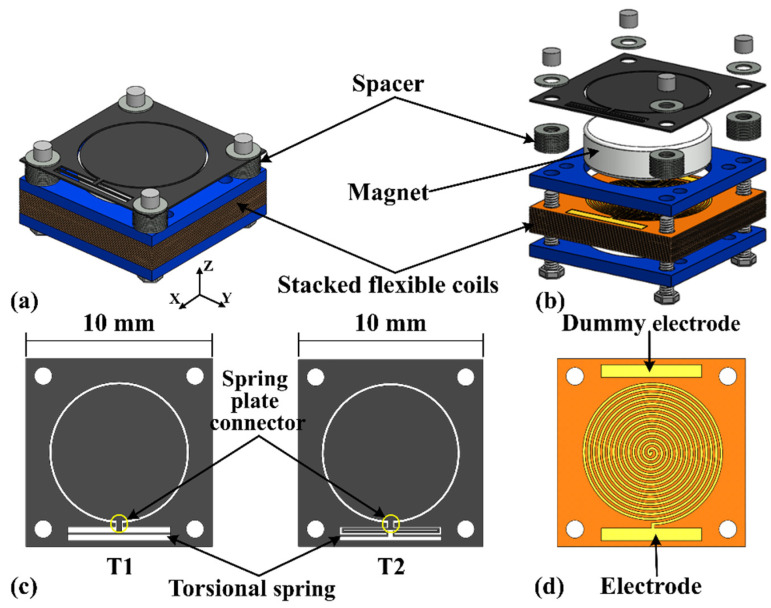
Schematic and exploded illustration of the assembled EVEH. (**a**) schematic illustration of the EVEH device; (**b**) exploded view of the EVEH device; (**c**) two types of torsional spring; (**d**) a layer of flexible coils.

The operation of the proposed EVEH device is based on the torsional movement of a disc magnet suspended by microfabricated torsional silicon springs, over a stack of high-density flexible coils, as shown in Figure 1a. Figure 1b shows the exploded view of the proposed EVEH device. A disc magnet is glued to the bottom surface of a silicon plate suspended by straight torsional springs. The magnetization direction of the disc magnet is along the z axis, as depicted by the coordinate in Figure 1a. Two types of torsional springs are presented in this work, i.e., the EVEH denoted as T1 consists of a straight torsional spring connecting to the magnet plate via a wide and short silicon beam (spring–plate connector), and device T2 consists of a folded torsional spring consists of three segments of straight springs, as shown in Figure 1c. For the fabrication of the silicon plate and torsional spring, a 200 μm thick monocrystalline silicon wafer is glued to a carrier wafer and etched through using inductively coupled plasma (ICP) reactive ion etching (RIE) process. A flexible coil stack is mounted below the disc magnet, and the coil–magnet distance is precisely controlled by the spacers between the coil and magnet (0.4 mm in this study). Each flexible coil layer consists of two oppositely wound spiral copper wires electroplated on the two sides of a 180 μm thick polyimide film, as shown in Figure 1d. The stack of flexible coils is aligned by the bolt holes and clamped together between two rigid FR4 layers, in order to be connected in series and form a coil with a large number of turns. Four M1 bolts are used to fix all the components. When the EVEH experiences external vibrations, the magnet oscillates torsionally (around the y axis in Figure 1) and generates electrical power in the coil. The torsional movement is mainly asserted on the long torsional beam, and the spring–plate connector will not deform due to its high stiffness. The main innovation of this technology is the torsional movement of the magnet and the formation of a high-density coil. The torsional movement of the magnet is capable of generating a large magnetic field gradient in the coils, with a reduced footprint. The overall footprint of the EVEH device is the same as the device reported in [20], but the functional area of the silicon layer (plate and spring area) is significantly reduced by using the torsional spring design. In this design, a much larger margin area is included in the device layer, which greatly facilitated the assembly process of the device. The stacked coils are formed by simple and low-cost repeating units of flexible coils. With proper technology development, the stacked coils may have the potential to be integrated into the fabrication of MEMS energy harvesters, enabling batch fabrication of the devices. The main design parameters of the EVEH device are listed in Table 1.

### 2.2. Modeling of the EVEH

According to Newton’s second law, the EVEH dynamic behavior can be described using a second-order spring mass damper system as follows:

(1) $$m\frac{d^{2}z\left(t\right)}{dt^{2}}+c\frac{dz\left(t\right)}{dt}+kz\left(t\right)=m\omega^{2}Y\sin\left(\omega t\right)$$

(2) $$2\pi f=\sqrt{\frac{k}{m}}$$

where *m* is the proof mass (magnet) of the EVEH device, *c* is the total damping coefficient including mechanical and electromagnetic damping, *k* is the effective stiffness of spring, *z*(*t*) is the relative displacement between the magnet and base of the z axis, *y*(*t*) = *Y*sin(*ωt*) is the external vibration applied to the base, and *f* is the resonant frequency of spring. According to Equation (2), the resonant frequency of the device can be reduced by decreasing the effective stiffness of the spring or increasing the mass of the magnet. For a torsional spring, the effective torsional stiffness can be described as follows [22]:

(3) $$T=\frac{KG\theta }{L}$$

where *K* is the spring torsion constant dependent on the form and dimensions of the cross section, *T* is the twisting moment, *L* is the length of the spring on y direction in Figure 1, *G* is the shear modulus of the spring, and *θ* is the torsional angle of spring (twisting around the y direction). According to [22], the value of *K* is given by

(4) $$K=ab^{3}\left[\frac{16}{3}-3.36\frac{b}{a}\left(1-\frac{b^{4}}{12a^{4}}\right)\right]\begin{array}{cc} \mathrm{for} & a\geq b \end{array}$$

where *a* and *b* are half of the width of the spring and half of the thickness of the spring, respectively. When the magnet moves under external vibration, according to Faraday’s law, the induced voltage in a coil within a changing magnetic field is given by

(5) $$V=-N\frac{d\phi }{dt}=-NA\frac{dB}{dz}\frac{dz}{d\theta }\frac{d\theta }{dt}$$

where *N* is the number of turns of the solenoid coil, *Φ* is the magnetic flux, *t* is time, *A* is the area of the coil, and *B* is the magnetic flux density. Additionally, when the EVEH device is twisting around the y direction (in Figure 1) at a small angle, the value of *z* equals *θ*. The magnetic flux density of a disc magnet along the central axis (z axis) can be described as follows [23]:

(6) $$B_{z}=\frac{B_{r}}{2}\left\{\frac{d+t}{\sqrt{{\left(d+t\right)}^{2}+R^{2}}}-\frac{d}{\sqrt{d^{2}+R^{2}}}\right\}$$

where *B_z_* is the magnetic flux density of the z axis, *B_r_* is the remnant magnetic flux density, *R* and *t* are the radius and thickness of the disc magnet, and *d* is the distance from the surface of the magnet. For a torsional spring, Equation (6) can be written as

(7) $$B_{z}=\frac{B_{r}}{2}\left\{\frac{r\sin\theta +d_{0}+t}{\sqrt{{\left(r\sin\theta +d_{0}+t\right)}^{2}+R^{2}}}-\frac{r\sin\theta +d_{0}}{\sqrt{{\left(r\sin\theta +d_{0}\right)}^{2}+R^{2}}}\right\}\cos\theta$$

where *r* is the distance between the center of the magnet and torsional spring, and *d*_0_ is the initial distance between magnet and coil. Figure 2 shows the analytical calculated magnetic flux density along the z-axis as a function of *θ*, and the magnetic field gradient (d*B*/d*θ*) is linear at a small angle.

Equation (4) indicates that under a certain external vibration, the following parameters can be increased in order to enhance the induced voltage: the number of coil turns (*N*), the area of the coil (*A*), the magnetic field gradient (d*B*/d*θ*) around the coil, or the torsional spring design (d*θ*/d*t*). However, when designing a miniaturized EVEH device, the coil area (*A*) and magnetic flux gradient (d*B*/d*θ*) are always limited by the device design (size and materials) and fabrication technologies. Therefore, a more effective way to increase induced voltage is by increasing the number of turns in the coil and the vibration amplitude of the EVEH device’s movable part. The vibration amplitude can be improved by different spring designs. In comparison, the number of coil turns (based on the planar coil technology) is difficult to improve in most of the reported MEMS EVEH devices.

**Figure 3:**
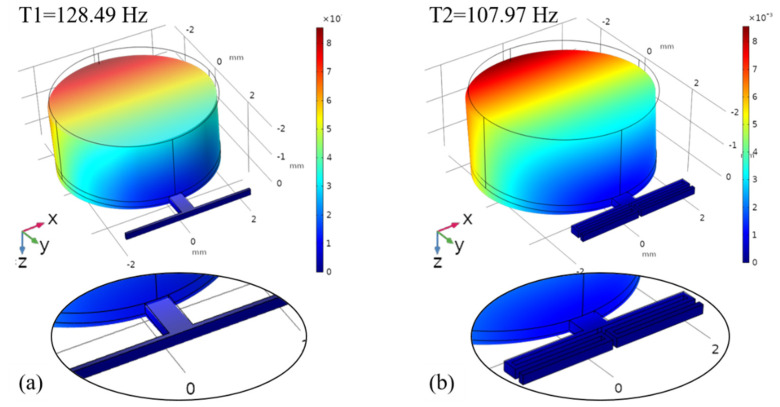
FEM modal analysis of the EVEH: (**a**) T1 device (128.49 Hz); (**b**) T2 device (107.97 Hz).

In order to study the dynamic characteristics of the EVEH device with a torsional spring, a FEM modal analysis of the proposed EVEH was performed with COMSOL Multiphysics software, as shown in Figure 3. In the FEM model, the simplification of the anchoring frame structure was carried out by applying fixed constraints to the end of the springs. This simplification is valid as the torsional spring is an in-plane spring. The material parameters of the silicon used in the FEM model were Young’s Modulus of 170 GPa and Poisson’s ratio of 0.28. The torsional vibration mode (the main resonance mode during operation, as the device is subjected to a uniform z-direction acceleration, and other resonant modes have a very low possibility be excited with the z-axis vibration) of the EVEHs is shown in Figure 3: T1 device twisting mode around the x-axis at 128.49 Hz, and T2 device twisting mode around the x-axis at 107.97 Hz. However, the FEM simulation of the torsional mode is not accurate, for the mode is influenced by the residual stress of the spring, and the complex stress distribution is difficult to include in the FEM model. The factor also ignored in the FEM model is the slight geometrical asymmetry caused by the device assembly process (e.g., the eccentric position of the magnet caused by the gluing process).

**Figure 4:**
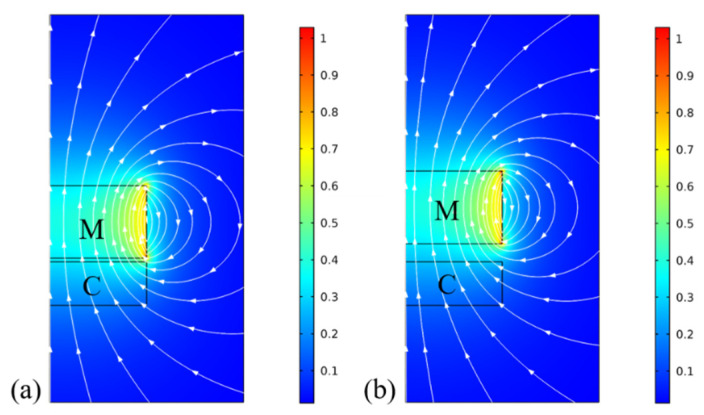
The magnetic flux of the magnet–coil structure based on a 2D axisymmetric FEM model at different magnet–coil distances: (**a**) 0.15 mm; (**b**) 0.75 mm.

Figure 4 shows the distribution of the magnetic flux over the coil at different magnet–coil distances: (a) 0.15 mm and (b) 0.75 mm. It was obtained from a 2D axisymmetric FEM model of the simplified magnet–coil structure. In the figure, M and C represent the magnet and coil, respectively, and the arrowed lines indicate the magnetic flux lines of the magnet with z-axis magnetization. When the distance between magnet and coil is increased from 0.15 mm to 0.75 mm, the magnetic flux density along the z axis is decreased from 0.34 T to 0.29 T. This change of the magnetic flux in the coil represents the origin of the induction process of the EVEH under vibration excitation.

## 3. Experimental

### 3.1. Assembly Process

**Figure 5:**
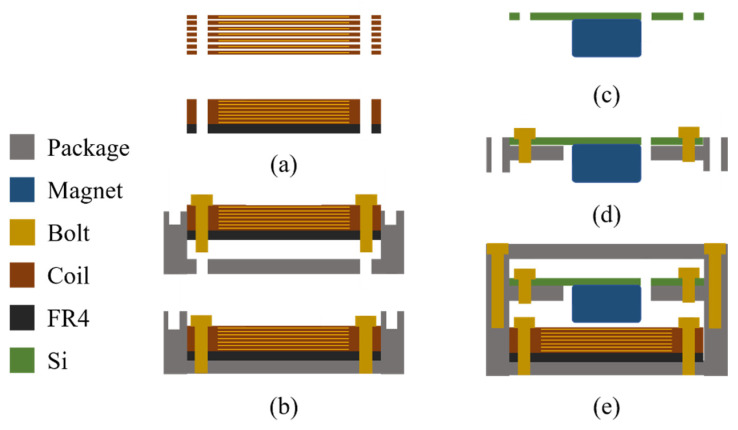
Assembly process of: (**a**) the stacked flexible coils; (**b**) the bottom package; (**c**) the magnet and device layer; (**d**) the intermediate package; (**e**) the package top cover.

Figure 5 shows the assembly process of the proposed EVEH. As shown in Figure 5a, the flexible coil layers were stacked onto a rigid FR4 frame, which contained electrical feedthroughs on the bottom. Subsequently, four bolts were used to fix the stacked coils and bottom package, as shown in Figure 5b. Afterward, the disc magnet was glued to a circular silicon plate connected to the torsional spring, as shown in Figure 5c. The intermediate package and the silicon layer device were then installed with four bolts, as shown in Figure 5d. Finally, the top cover of the package was installed by using four bolts, as shown in Figure 5e. The package of the EVEH device in this work was an anodized aluminum case for both mechanical protection and electrical insulation.

### 3.2. Measurement Setup

**Figure 6:**
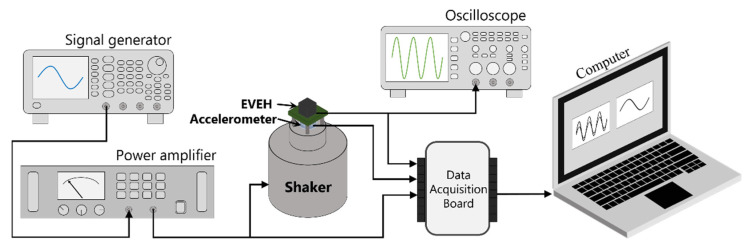
Schematic illustration of the measurement setup.

Figure 6 shows the measurement setup for characterizing the dynamic behavior of the fabricated EVEH. In the setup, the EVEH device was mounted on a vibration shaker, which was excited by a sinusoidal signal produced by the signal generator and amplified by a power amplifier. The real-time excitation acceleration was monitored by an accelerometer mounted underneath the EVEH by a customized installation fixture. Experimental data including the EVEH output and applied acceleration signals were collected simultaneously by a NI USB-6211 data acquisition board and LabVIEW software, with a sampling rate of 1000 samples per second. A fourth-order Butterworth low pass filter with a cut-off frequency of 500 Hz was used to remove the high-frequency noise, implemented in the LabVIEW software. The acquired voltage in this work has a measurement error of ±0.05 mV, derived from the standard deviation of 594 periods of sinusoidal voltage output.

## 4. Results and Discussions

### 4.1. Fabrication Results of the EVEH Device

**Figure 7:**
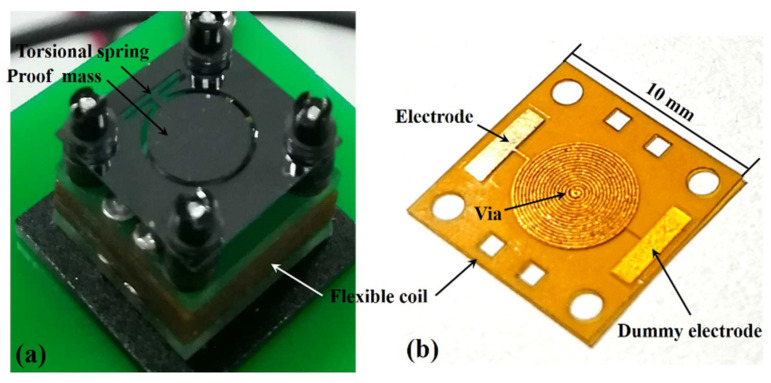
Photos of (**a**) assembled EVEH device (T1) and (**b**) a layer of the flexible coil.

The designed EVEH device (T1 device) was successfully fabricated and assembled, as shown in Figure 7a. Additionally, for the T2 device, the difference is the spring type shown in Figure 1c. The NdFeB disc magnet was glued to the bottom of the circular silicon microplate as the proof-mass (not visible in the photo). The magnet proof-mass was suspended by a long torsional beam above a stack of flexible coils. Under external vibration excitation, the relative position of the magnet and the coil changed to generate a changing magnetic field, and an electrical voltage was induced in the coil. The stacked flexible coils (clamped between two rigid FR4 frames) were mounted on the bottom of the EVEH device. Additionally, for a large number of turns, the thickness of the stacked flexible coils is reasonable. The thin substrates make it possible to have high-density coils close to the magnet. This is essential for the miniaturized device, as the magnetic field decays exponentially from the outer surface of the magnet. Through this coil design, the EVEH device can achieve high output power with a minimum footprint. The proposed technology is a general technology that can address the limitations of planar coils widely used in MEMS devices. This method is simple and cost effective because the coil stack is composed of repeating low-cost flexible layers. In addition, the distance between the magnet and the coils could be adjusted by the spacers between them. The flexible coil had an identical spiral coil on each side but with opposite winding directions, connected by the via in the center of the coil, as shown in Figure 7b. The electrode and dummy electrode were located at the edge of the film, and the positions were reversed on the other side of the coil layer. When two layers were clamped together (the electrodes were connected), a coil with four windings connected in series was formed. The dummy electrodes were patterned on each side of the coil layer to maintain the mechanical symmetry of the coil layer during stacking and clamping. For comparing the performance of four types of spring design, the coil size and the overall footprint of the EVEH in this work is the same as the device reported in [20], but the functional area of the silicon layer is greatly reduced, which has the potential to the goal of miniaturization.

### 4.2. Open-Circuit Frequency Domain Response of the EVEH Device

**Figure 8:**
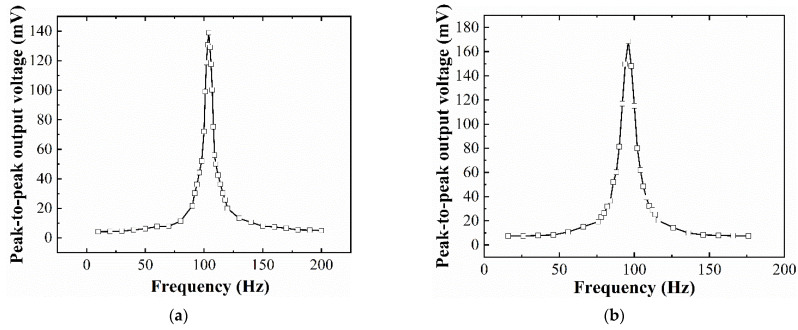
The peak-to-peak open-circuit output voltage of the EVEH as a function of frequency: (**a**) T1 device; (**b**) T2 device.

Figure 8 shows the open-circuit peak-to-peak output voltage of the assembled EVEH device as a function of frequency, under a sinusoidal excitation (the most common vibration signal existing in both nature and industry), with an amplitude of ±0.5 g. The output voltage of the EVEH increases gradually as the frequency of the excitation vibration increases toward the resonant frequency, reaches a maximum value at resonance and then decreases again as the frequency further increases. For device T1, at the resonant frequency of 104 Hz, the output voltage reaches the maximum peak-to-peak value of 138.9 mV. For device T2, the maximum open-circuit peak-to-peak output voltage is 169 mV at the resonant frequency of 96 Hz. It is known that most of the ambient vibrations concentrate in the frequency band of 1–200 Hz, the resonant frequency around 100 Hz of the devices T1 and T2 may find applications in several industrial scenarios such as power transformers, transmission lines, and power inductors. In comparison, the open-circuit peak-to-peak output voltage of the EVEH devices presented in [20] is 208.3 mV at the frequency of 143 Hz for D1 and 149.3 mV at the frequency of 156 Hz for D2. Additionally, the functional area of T1, T2, D1, and D2 are 20.3 mm^2^, 20.6 mm^2^, 23.4 mm^2^ and 24.1 mm^2^, respectively. Thus, the torsional springs with a reduced functional area of the silicon layer and lower frequency generate a higher output voltage.

The presented energy harvester (T1 device) achieves 10 times higher maximum output voltage at 3.8 times lower resonant frequency, compared with a two-degree-of-freedom EVEH based on coils suspended by springs [24], and almost 188 times higher maximum output voltage at 1.2 times lower resonant frequency, compared with an EVEH based on magnet suspended by folded springs reported in 2014 [25]. Compared with the traditional planar coil used in MEMS or PCB technologies, the optimized performance of the EVEH device results from the stacked flexible coils, which greatly enhance the number of turns of the coils.

### 4.3. Impedance Matching

To study the optimal closed-circuit behavior of the EVEH, matching resistors between 1 Ω and 1000 Ω were connected to the output of the energy harvester. Through the measured closed-circuit voltage, the output power of the EVEH can be described as

(8) $$P=\frac{V_{rms}^{2}}{R_{L}}=\frac{V_{pp}^{2}}{8R_{L}}$$

where *V_rms_* is the root-mean-square voltage over the matching resistor, *R_L_* is the resistance of the matching resistor, and *V_pp_* is the peak-to-peak voltage over the matching resistor.

**Figure 9:**
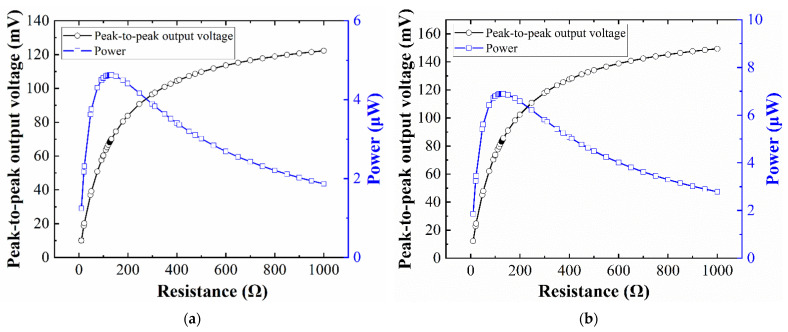
The peak-to-peak output voltage and power of the EVEH as a function of load resistance: (**a**) T1 device; (**b**) T2 device.

Figure 9 shows the closed-circuit peak-to-peak output voltage and output power of the EVEH device as a function of the load resistance, under a sinusoidal excitation with an amplitude of ±0.5 g and frequency of 104 Hz for T1 device (96 Hz for T2 device). For both types of devices, the internal (coil) resistance of the EVEH under test is 128.7 Ω. With the increase in load resistance, the output voltage of the EVEH first increases sharply and then decreases gradually. For the T1 device, the maximum power measured is 4.6 μW at the load resistance of 128.7 Ω and the resonant frequency of 104 Hz. In comparison, the T2 device has a maximum power measured as 6.9 μW at the load resistance of 128.7 Ω and the resonant frequency of 96 Hz. Additionally, with the same coil resistance, both designs of the proposed torsional EVEHs have lower frequencies than previous work in [20]; the maximum output power is 10.5 μW (143 Hz) for the D1 device and 5.4 μW (156 Hz) for the D2 device.

### 4.4. Closed-Circuit Frequency-Domain Response of the EVEH Device

**Figure 10:**
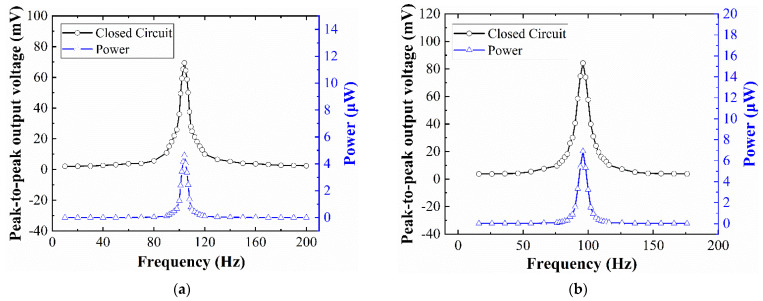
The peak-to-peak closed-circuit output voltage and power of the EVEH device as a function of frequency: (**a**) T1 device; (**b**) T2 device.

Figure 10 shows the closed-circuit peak-to-peak output voltage and output power of the EVEH device when connected to a load resistance of 128.7 Ω. The measurements were performed under sinusoidal excitation of ±0.5 g. For the T1 device, the maximum peak-to-peak closed-circuit output voltage is 69.4 mV at 104 Hz (resonant frequency). For device T2, the maximum peak-to-peak closed-circuit output voltage of the EVEH is 84 mV at 96 Hz (resonant frequency). Due to the similar values of the coil and load resistance, the closed-circuit output voltage is approximately half of the open-circuit voltage in Figure 8. The maximum power device T1 and T2 are 4.6 μW at the frequency of 104 Hz and 6.9 μW at the frequency of 96 Hz, respectively. The performance of the EVEH device is improved, compared with recently reported work with similar technologies, in terms of output voltage, power, and resonant frequency (low resonant frequency is preferable for matching to the low-frequency ambient vibrations). For example, Zhang et al. reported an EVEH based on MEMS technology with an output power of 0.82 μW at the resonant frequency of 350 Hz under the acceleration of 4.5 g [26]. Tao et al. reported a MEMS electromagnetic vibration energy harvester with two degrees of freedom; the maximum amplitude of the output voltage in the study is 6.5 mV at the resonant frequency of 391 Hz [24]. The maximum closed-circuit output power of 4.6 μW (T1 device) is 4792 times higher than the maximum closed-circuit output power of 0.96 nW with an optimum load resistance of 12 kΩ at 0.12 g vibration reported in [24]. Since the volume of the EVEH device and external excitation vibration (*A*_0_) have a greater impact on device performance, to accurately describe it, the normalized power density (NPD) is given by

(9) $$\mathrm{NPD}=\frac{P}{\mathrm{Volume}\cdot A_{0}^{2}}$$

The calculated NPD values of the T1 device and T2 device are 17.04 (μW/cm^3^/g^2^) and 25.56 (μW/cm^3^/g^2^), respectively. Additionally, the NPD value is 38.89 (μW/cm^3^/g^2^) for D1 device and 20 (μW/cm^3^/g^2^) for D2 device [20]. Tao et al. reported a MEMS electromagnetic vibration energy harvester with two degrees of freedom in 2016 with the NPD value of up to 0.23 (μW/cm^3^/g^2^) at the resonant frequency of 391 Hz from 0.02 to 0.2 g [24]. Liu et al. reported an in-plane approximated nonlinear MEMS electromagnetic energy harvester in 2014 with the NPD value of 0.62 × 10^−2^ (μW/cm^3^/g^2^) at the resonant frequency of 146.5 Hz and acceleration of 3 g [25]. Compared with the work reported in [24], the coil resistance in this work is much smaller, so the increase in closed-circuit output power is much bigger than the square of the increase in open-circuit output voltage (see discussions about Figure 8). In addition, with the same footprint, the device T2 has increased its NPD by 50%, compared with T1, indicating the good potential of optimization for the torsional design of the EVEH device. For future optimization of the springs, coils, and magnets, the output power of the proposed EVEH device can be further enhanced.

### 4.5. Acceleration Test

**Figure 11:**
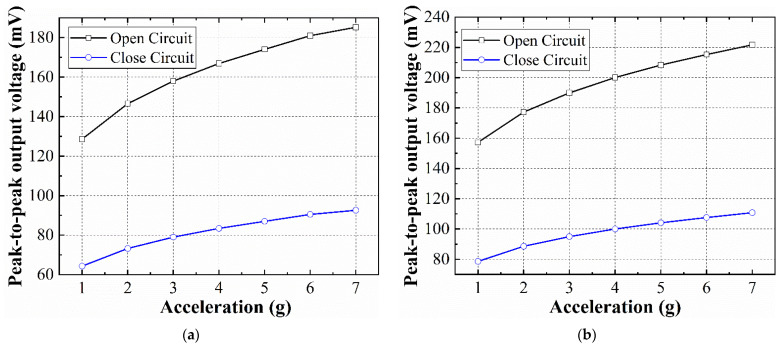
The peak-to-peak output voltage measured in the open circuit and closed circuit at different accelerations: (**a**) T1 device; (**b**) T2 device.

The dependence of the EVEHs output on the acceleration level was studied, and the open-circuit and closed-circuit peak-to-peak output voltages of the EVEH device were measured as a function of acceleration, as shown in Figure 11. All data were obtained at EVEH’s resonant frequency of 104 Hz, for the T1 device, and 96 Hz for the T2 device; the applied peak-to-peak acceleration increases from 1 g to 7 g (step length is 1 g). At acceleration higher than 7 g, the distance between coil and magnet must be increased to reduce the potential collision. The magnet–coil distance can be increased by simply changing the thickness of the spacers. At an acceleration of 7 g, the maximum peak-to-peak open-circuit output voltage is 185.2 mV and 222 mV for device T1 for T2, respectively. Compared with a previous work, also based on microfabricated springs, where the maximum peak-to-peak open-circuit output voltage is 333.1 mV and 225.5 mV at 7 g [20], the output voltage–acceleration relationship in this work shows a similar trend but lower maximum voltage at higher acceleration levels. The output voltage in [20] increases almost linearly as the acceleration increases, whereas the output voltage of devices shows a gradually decreasing rate of enhancement as the acceleration increases. The possible explanation for this phenomenon is that the shear strain induced in the single-beam torsional spring is much larger than the shear strain in the multibeam folded spring, at the same acceleration. Therefore, at elevated acceleration, nonlinearity in the torsional spring arises and limits the deflection angle of the magnet, which reduces the amplitude of enhancement of the output voltage. In addition, it is increasingly difficult for the magnet to maintain its movement perpendicular to the coil plane (z direction) as the vibration excitation acceleration increases, and there will be a tendency to twist in the x or y direction. The torsional vibration tendency will reduce the working efficiency of the device, thereby reducing the output voltage of the device. The closed-circuit output voltage shows a similar linear characteristic to the open-circuit output but with half of the amplitude of the open-circuit output.

## 5. Conclusions

In this work, an EVEH device was designed and fabricated based on hybrid PCB and MEMS technology. The proposed EVEH was composed of a stack of flexible coils and a disc permanent magnet mounted vertically. The assembled EVEH was excited by sinusoidal accelerations and its dynamic behavior was characterized. At a sinusoidal acceleration with a peak-to-peak value of 1 g (±0.5 g, ±4.9 m/s²), a maximum output peak-to-peak voltage of 169 mV and output power of 6.9 μW are realized at a resonant frequency of 96 Hz. At an elevated acceleration of 7 g (±3.5 g), a maximum output peak-to-peak voltage of 222 mV is realized. In the future, the bonding technologies between the stacked coils and silicon proof mass will be explored.

## Figures and Tables

**Figure 2:**
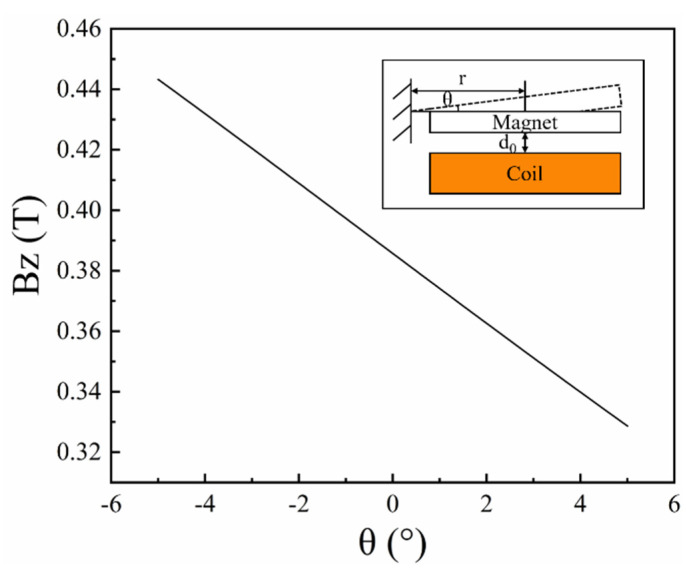
Curve of magnetic flux density along the z axis as a function of *θ.*

**Table 1:** Main design parameters of the EVEH device.

Parameters		Designed	Measured
Overall device	Size of device (L × W × H) (mm)	10 × 10 × 10.7	10 × 10 × 10.8
	Thickness of the wafer (mm)	0.2	0.2
Device T1	Size of torsional spring (L × W) (mm)	4 × 0.08	4 × 0.08
	Size of spring–plate connector (L × W) (mm)	0.4 × 0.8	0.4 × 0.8
	Functional area (mm^2^) *	20.3	20.3
Device T2	Size of torsional spring unit (L × W) (mm)	2.04 × 0.08	2.04 × 0.08
	Number of torsional spring folds	2	2
	Size of spring–plate connector (L × W) (mm)	0.5 × 0.4	0.5 × 0.4
	Functional area (mm^2^) *	20.6	20.6
Magnet	Diameter (mm)	4.00	4.04
	Thickness (mm)	3.00	3.04
Coil	Numbers of turns	40	40
	Magnet–coil distance (mm)	0.40	0.45

* Functional area indicates the total area occupied by the silicon plate and springs.

## Data Availability

The data presented in this study are available in [insert article or supplementary material here].
